# Effectiveness of wearable devices as a support strategy for maintaining physical activity after a structured exercise intervention for employees with metabolic syndrome: a randomized controlled trial

**DOI:** 10.1186/s13102-022-00409-1

**Published:** 2022-02-10

**Authors:** Pauline Bayerle, Arno Kerling, Momme Kück, Simone Rolff, Hedwig Theda Boeck, Thorben Sundermeier, Ralf Ensslen, Uwe Tegtbur, Dirk Lauenstein, Dietmar Böthig, Christoph Bara, Alexander Hanke, Christoph Terkamp, Axel Haverich, Meike Stiesch, Martina de Zwaan, Sven Haufe, Lars Nachbar

**Affiliations:** 1 Institute of Sports Medicine, Hannover Medical School, Carl-Neuberg-Str. 1, 30625 Hannover, Germany; 2 Department of Psychosomatic Medicine and Psychotherapy, Hannover Medical School, Hannover, Germany; 3 Volkswagen AG, Wolfsburg, Germany; 4 Audi BKK Health Insurance, Ingolstadt, Germany; 5 Department of Cardiac, Thoracic, Transplantation and Vascular Surgery, Hannover Medical School, Hannover, Germany; 6 Department of Gastroenterology, Hepatology and Endocrinology, Hannover Medical School, Hannover, Germany; 7 Department of Prosthetic Dentistry and Biomedical Materials Science, Hannover Medical School, Hannover, Germany

**Keywords:** Physical activity, Telemonitoring, Wearable device, Metabolic syndrome, Sustainability, Maintenance, Company employees, Work ability

## Abstract

**Background:**

Metabolic syndrome (MetS) is associated with an increased risk for cardiovascular events and high socioeconomic costs. Despite lifestyle interventions focusing on exercise are effective strategies to improve parameters of the above aspects, many programs fail to show sustained effects in the long-term.

**Methods:**

At visit 2 (V2) 129 company employees with diagnosed MetS, who previously participated in a 6-month telemonitoring-supported exercise intervention, were randomized into three subgroups for a 6-month maintenance treatment phase. A wearable activity device was provided to subgroup A and B to assess and to track physical activity. Further subgroup A attended personal consultations with individual instructions for exercise activities. Subgroup C received neither technical nor personal support. 6 months later at visit (V3), changes in exercise capacity, MetS severity, work ability, health-related quality of life and anxiety and depression were compared between the subgroups with an analysis of variance with repeated measurements.

**Results:**

The total physical activity (in MET*h/week) declined between visit 2 and visit 3 (subgroup A: V2: 48.0 ± 33.6, V3: 37.1 ± 23.0; subgroup B: V2: 52.6 ± 35.7, V3: 43.8 ± 40.7, subgroup C: V2: 51.5 ± 29.7, V3: 36.9 ± 22.8, for all p = 0.00) with no between-subgroup differences over time (p = 0.68). In all three subgroups the initial improvements in relative exercise capacity and MetS severity were maintained. Work ability declined significantly in subgroup C (V2: 40.3 ± 5.0, V3: 39.1 ± 5.7; p < 0.05), but remained stable in the other subgroups with no between-subgroup differences over time (p = 0.38). Health-related quality of life and anxiety and depression severity also showed no significant differences over time.

**Conclusions:**

Despite the maintenance of physical activity could not be achieved, most of the health related outcomes remained stable and above baseline value, with no difference regarding the support strategy during the maintenance treatment phase.

*Trial registration* The study was completed as a cooperation project between the Volkswagen AG and the Hannover Medical School (ClinicalTrials.gov Identifier: NCT02029131).

## Background

The metabolic syndrome (MetS) is a cluster of interrelating metabolic risk factors consisting of abdominal obesity with elevated waist circumference, insulin resistance, dyslipidemia and elevated blood pressure [1]. It is estimated that the metabolic syndrome is present in around 20–25% of the world’s adult population and is considered the driving force for a new cardiovascular disease epidemic [2].

People with MetS have a fivefold greater risk of developing type 2 diabetes, which consequently leads to significant higher health care costs and socioeconomic expenses [3]. In 2019, 10% of global health expenditure (760 billion US dollar) is spent upon diabetes. If diabetes prevalence continues to rise as anticipated, 700 million people worldwide will live with type 2 diabetes by 2045 [4]. Further unfavorable outcomes like cardiovascular events, an approximately twice-increased risk of incident cardiovascular mortality and certain cancer types are also linked with the MetS [5, 6].

Reducing risk factors of the MetS on the other hand is associated with reduced health care, pharmacy and short-term disability expenses as well as increased productivity outcomes [7].

The evidence characterizing the benefits of physical activity (PA) in general [8] and in people with MetS specifically is extensive [9]. Yet, in 2016, globally more than 25% of all adults and 36.8% of the population in high-income Western countries show insufficient PA [10]. Lifestyle interventions, including supervised exercise programs, are key components in the treatment of patients with MetS [11, 12], but many exercise programs fail to show sustained gains in the long-term. Previous studies have shown that lifestyle intervention programs based on both nutritional counselling and physical exercise are effective in patients with MetS, but likewise effects diminish over time [12, 13]. In a systematic review by Lynch et al. including twenty-one clinical studies the results imply that wearable devices may enhance PA interventions by using self-monitoring as a meaningful technique for changing PA behavior [14]. Yet, there appears to be no consensus about what contributes to successful physical activity maintenance [15].

Recently, we were able to show that a 6-month telemonitoring-guided and wearable activity device-supported exercise intervention with the goal of 150 min of moderate activity per week not only reduced MetS severity, but also has a significant potential to reduce disease risk while also improving mental health, work ability, and productivity-related outcomes in company employees with diagnosed MetS [16].

To assess the long-term effects of this intervention and the potential sustainability of obtained results, participants of the exercise group were assigned to a 6-month maintenance treatment phase in which the originally applied type of intervention was maintained or reduced regarding to personal or technical support.

We hypothesized that the maintenance of personal and technical support, as initially applied is superior for maintaining physical activity-induced benefits compared to lower-intense support strategies.

## Material and methods

### Study design and participants

The study on which this work is based is divided into two parts, which are presented below as the initial intervention phase and the maintenance treatment phase. Initially, all participants took part in a prospective, randomized, parallel-group and single-blind (assessor blind) controlled trial examining the effects of telemonitoring-supported exercise training on MetS severity and work ability in company employees.

**Fig. 1:**
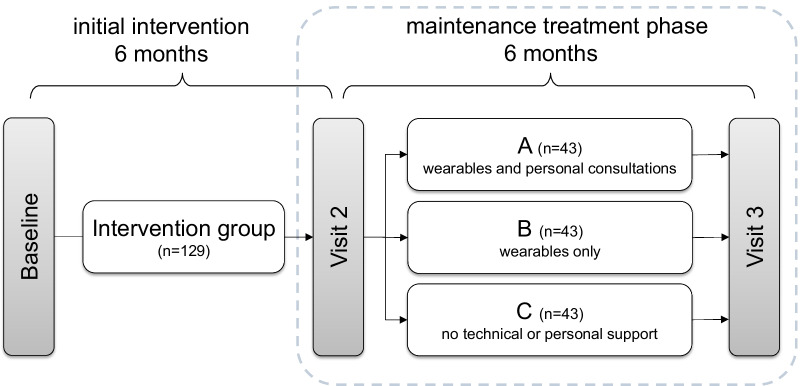
Study design

The study was done as a collaborative project between Volkswagen AG and Hannover Medical School and took place at the main Volkswagen factory in Wolfsburg, Germany. After the participants were recruited via a series of information events on the factory premises, the baseline visit started in October 2017, initializing the 6-month exercise intervention. The total of recruited participants (n = 314) consisted of 52% office workers and 36% manual workers (12% unclassified). 21% of the participants worked shifts, 72% were non-shift workers (7% unclassified) [16]. After 6 months at visit 2, participants of the exercise group (19 women, 110 men, age: 48.6 ± 7.6 yrs., BMI: 31.6 ± 4.7 kg/m^2^) with diagnosed MetS were randomized 1:3 into three subgroups (subgroup A, B and C). Visit 2, starting in April 2018, was the starting point for the maintenance treatment phase which lasted a further 6 months, with each subgroup undergoing a different support strategy during this phase (Fig. 1). The evaluation of the maintenance treatment phase took part at visit 3. The overall course of the study is shown in Fig. 1.

Subgroup A (n = 43, 5 women, 38 men) continuously used wearable devices (Forerunner 35, Garmin, Garching, Germany) with which activities and daily step count were recorded. As during the first intervention, the smartphone application Rebirth Active was used again. Additionally, subgroup A attended personal feedback consultations with individual instructions for exercise activities at month 2 and 4. An exercise scientist performed the individual counseling in order to keep everyday activity on a high level and to adjust the training schedule to participants’ current situation.

Subgroup B (n = 43, 8 women, 35 men) also received technical support from wearable devices, general information via the Rebirth Active application but no further personal consultations.

Subgroup C (n = 43, 6 women, 37 men) received neither technical support, for they had to hand in their wearable device after completing visit 2, nor individualized support by exercise scientists.

The randomization was computer-based generated by an external collaborator. Study nurses and physicians examining participants were blinded for the randomization sequence.

With the start of the initial intervention the exercise group was equipped with a wearable activity device worn on the wrist of the non-dominant hand. After a short introduction, participants were asked to wear the activity device throughout the intervention period. Daily step count was recorded continuously. In addition, sporting activities (eg, cycling, cardio training, walking outdoors, and walking indoors) were asked to be tracked. Activity time, distance, and heart rate ( assessed by an optical heart-rate sensor were recorded. Data was transferred to a server at the Hannover Medical School and was traced and assessed by the exercise scientists.

Right from the start, the aim was to keep everyday activity as high as possible and to meet the WHO target of at least 150 min of moderate activity per week. In order to support the participants in an active lifestyle, but also to individually adapt the training, monthly personal consultations were held on the factory premises. Additionally, general and individual recommendation on health issues, information about the study background and study target dates were communicated via the smartphone application Rebirth Active (d.velop AG, Gescher, Germany).

After the baseline visit as well as after visit 2 and 3, the participants in the exercise group or each subgroup received personal nutritional advice upon a 7-day food diary and general information on healthy nutrition based on the general recommendations of the German Society for Nutrition (https://www.dge.de/index.php?id=52).

According to baseline visit, we distributed questionnaires to collect data on health-related quality of life (short form 36 [SF-36]) [17], on anxiety and depression severity (HADS-D) [18] and on work ability (work ability index [WAI]) [19] at visit 2 and 3. To calculate the total and exercise-related PA metabolic equivalents of task as (MET)-hours per week the Freiburger Physical Activity questionnaire was applied [20]. The HADS-D is composed of fourteen items that cluster into two subscales for anxiety and depression. The score range lies within 0 to 21 points, whereby a higher score indicates more severe symptomatic. The SF-36 consists of a mental- and physical component score. For both sum scores, the score range lies within 0 to 100, with 100 points representing the maximum quality of life (QoL). The WAI questionnaire contains questions concerning work, work ability and health with one total score ranging from 7 (minimum) to 49 (maximum) points.

Further examinations including a general medical examination by a physician (including electrocardiogram, case history, and physical examination) and measurement of bodyweight, waist circumference and height were executed in a standardized way. Body-mass-index (BMI) was calculated with the formula bodyweight (kg) ÷ height (m2).

Fat-free mass and fat mass as markers of body composition were estimated by segmental multi-frequency bioimpedance analysis (InBody720; Biospace, South Korea). Blood pressure was measured after 5 min of rest with a suitable automatic blood pressure cuff (Critikon, Dinamap, USA) as the mean value of two successive recordings.

All examinations were identical on each visit. The description of the measured values from blood samples as well as the calculation of the MetS severity score (MetS-z-score) is described in detail previously [16].

Based on maximum workload and heart rate during the exercise ergometry at visit 2 (Schiller 911 BPplus, Schiller, Feldkirchen, Germany), participants were advised to continue their exercise training established during the initial intervention phase at an individually recommended heart rate. The given task throughout the maintenance treatment phase was to keep PA on a high level, targeting the WHO guidelines of minimum 150 min of moderate activity per week [21]. To evaluate participants’ adherence to these guidelines, the duration of recorded PA with wearable devices were used. The outcomes of the Freiburger Physical Activity questionnaire in terms of everyday, exercise and total PA were used as an aid to evaluate and compare self-reported PA over time and between the subgroups.

### Statistical analysis

The analysis for all outcomes was done in the intention-to-treat population, including all patients, who were randomized at visit 2. Missing values were replaced by the Last Observation Carried Forward method. All values are shown as mean ± standard deviation. The normal distribution was tested with a Shapiro–Wilk-test. To test distribution a Chi-squared test was used. Differences between the three groups at visit 2 were calculated with a one-way ANOVA for parametric data or a Kruskal–Wallis-Test for non-parametric data, respectively. Differences within a group over time were calculated with a paired t-Test for parametric data respectively a Wilcoxon test for non-parametric data. The interaction between time and group and differences across three time points within the whole intervention group were tested with an analysis of variance with repeated measurements with η^2^ as effect size. Post hoc tests were corrected according to Bonferroni. Data were analyzed using SPSS for Windows (SPSS 26, IBM, Armonk, NY, USA). We considered p < 0.05 as statistically significant.

## Results

**Fig. 2:**
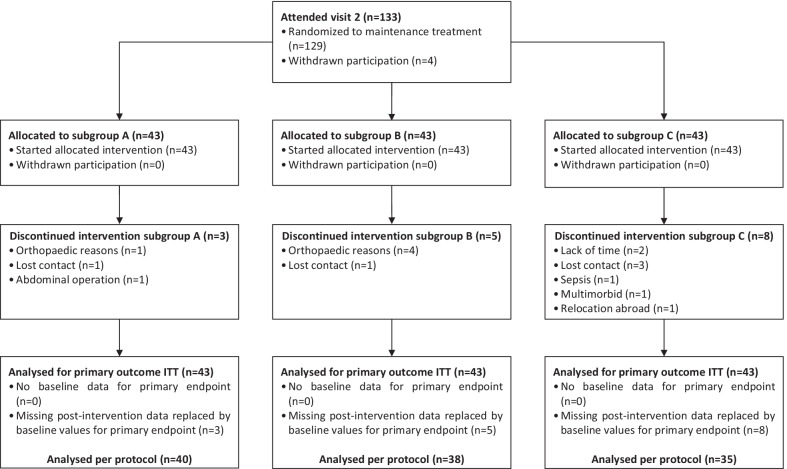
Allocation of participants at maintenance treatment phase including dropouts

In 113 out of 129 subjects, data for visit 3 was available. Across all three subgroups, there have been 16 recorded dropouts during the maintenance treatment phase. Four subjects were not randomized for they have withdrawn their participation after visit 2 without giving reasons (Fig. 2).

At visit 2, the three subgroups did not differ regarding sex, age, body composition or the five MetS components (Table 1).

**Table 1:** Subject characteristics at visit 2

Parameter	A	B	C	p-value
Female / male	5 / 38	8 / 35	6 / 37	0.649
Age (yrs)	50.3 ± 7.2	47.9 ± 8.2	47.6 ± 7.5	0.194
Body weight (kg)	102.8 ± 19.0	99.7 ± 15.9	99.8 ± 16.8	0.638
Body mass index (kg/m^2^)	32.0 ± 5.4	31.4 ± 4.7	31.3 ± 4.5	0.742
Waist circumference (cm)				
Female	106.6 ± 8.3	107.6 ± 11.0	102.7 ± 8.2	0.623
Male	111.1 ± 13.1	107.9 ± 12.2	108.3 ± 12.4	0.481
Fasting glucose concentration (mg/dl)	111.3 ± 35.8	99.1 ± 15.7	102.9 ± 17.5	0.071
Triglyceride (mg/dl)	180.6 ± 207.8	153.3 ± 94.9	191.8 ± 140.0	0.491
HDL (mg/dl)	44.5 ± 8.9	47.5 ± 8.6	43.5 ± 9.5	0.111
Resting RR_sys_ (mmHg)	129.9 ± 10.1	129.5 ± 11.8	133.2 ± 11.3	0.140
MetS-z-score	0.75 ± 0.85	0.47 ± 0.64	0.72 ± 0.71	0.232

Subgroup A—wearables and personal consultation, Subgroup B—wearables only, Subgroup C—no technical or personal support

*HDL* high density lipoprotein

**Fig. 3:**
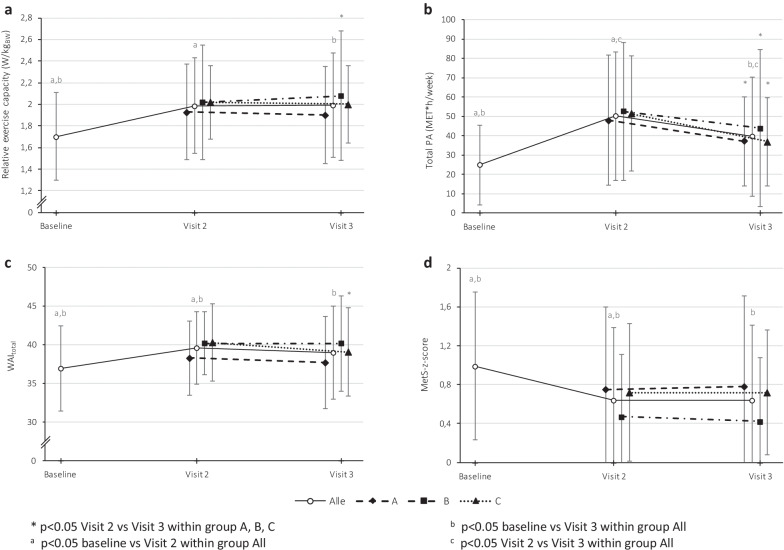
**a**–**d** Relative exercise capacity (**a**), total PA (**b**), WAI_total_ (**c**) and MetS-z-score (**d**) in the whole intervention group across baseline, visit 2 and visit 3, and in the subgroups A, B and C across the maintenance phase from visit 2 to visit 3. Relative exercise capacity (p < 0.001, η^2^ = 0.46), total PA (p < 0.001, η^2^ = 0.24) and WAI_total_ (p < 0.001, η^2^ = 0.18) increased significantly across the three time points, respectively decreased for MetS-z-score (p < 0.001, η^2^ = 0.23). *WAI* work ability index, *PA* physical activity

As one of the major outcomes, relative exercise capacity (W/kg_BW_) increased significantly for the whole intervention group between baseline and visit 2 and remained above baseline at visit 3 (Fig. 3a). After 6-month maintenance treatment phase, relative exercise capacity remained stable for subgroup A (wearables and personal consultation) and C (no technical or personal support) and increased significantly for subgroup B (Fig. 3a).

Similar findings for the parameters total physical activity (MET*hours/week) (Fig. 3b), work ability (WAI) (Fig. 3c) and MetS-z-score (Fig. 3d) were observed: The results at visit 3 remained above baseline value for the whole intervention group.

Among the MetS components, the systolic blood pressure in subgroup A (wearables and personal consultations) and waist circumference in subgroup C (no technical or personal support) was worsened at visit 3. No significant differences in other MetS components or in the MetS-z-score itself were observed (Table 2).

**Table 2:** Change after 6 months maintenance treatment phase

	A		B		C		Time	Time × group	
	Visit 2	Visit 3	Visit 2	Visit 3	Visit 2	Visit 3	p-value	p-value	η^2^
Body weight (kg)	102.8 ± 19.0	103.9 ± 19.4*	99.7 ± 15.9	99.5 ± 16.6	99.8 ± 16.8	100.3 ± 17.8	0.116	0.148	0.03
Body mass index (kg/m^2^)	32.0 ± 5.4	32.4 ± 5.6*	31.4 ± 4.7	31.4 ± 5.0	31.3 ± 4.5	31.4 ± 4.8	0.122	0.157	0.03
Fat mass (kg)	31.7 ± 13.5	32.9 ± 13.4*	30.5 ± 11.5	30.5 ± 12.7	30.5 ± 12.3	30.6 ± 12.8	0.143	0.299	0.02
Fat-free mass (kg)	71.3 ± 9.7	71.0 ± 10.1	69.1 ± 12.4	69.3 ± 13.0	69.4 ± 10.3	69.7 ± 11.0	0.862	0.652	0.01
Body fat (%)	29.7 ± 8.6	30.7 ± 8.3*	30.2 ± 9.7	30.2 ± 10.3	30.0 ± 8.6	29.8 ± 9.0	0.341	0.110	0.03
Waist circumference (cm)	110.6 ± 12.6	112.0 ± 13.9	107.8 ± 11.9	107.6 ± 12.8	107.5 ± 12.0	109.0 ± 12.5*	0.042	0.176	0.03
Glucose (mg/dl)	111.3 ± 35.8	110.8 ± 30.3	99.1 ± 15.7	99.4 ± 16.5	102.9 ± 17.5	100.7 ± 13.3	0.560	0.745	0.00
Triglyzeride (mg/dl)	180.6 ± 207.8	189.1 ± 218.2	153.3 ± 94.9	140.3 ± 80.5	191.8 ± 140.0	187.7 ± 131.9	0.630	0.341	0.02
HDL (mg/dl)	44.5 ± 8.9	44.7 ± 9.9	47.5 ± 8.6	46.4 ± 10.0	43.5 ± 9.5	43.0 ± 8.7	0.299	0.532	0.01
RR_sys_ (mmHg)	129.9 ± 10.1	134.1 ± 12.5*	129.5 ± 11.8	129.7 ± 11.0	133.2 ± 11.3	134.8 ± 14.6	0.042	0.256	0.02
RR_dia_ (mmHg)	83.2 ± 8.7	86.3 ± 9.9*^a^	84.7 ± 9.2	82.5 ± 7.1^a^	85.5 ± 6.8	86.3 ± 9.1	0.405	0.006	0.08
MetS-z-score	0.75 ± 0.85	0.78 ± 0.93	0.47 ± 0.64	0.42 ± 0.66	0.72 ± 0.71	0.72 ± 0.64	0.862	0.676	0.01
Peak exercise capacity (W)	193.5 ± 35.4	192.1 ± 36.4	197.7 ± 45.9	203.0 ± 51.8*	198.8 ± 32.9	197.9 ± 35.8	0.532	0.164	0.03
Relative exercise capacity (W/kg_BW_)	1.93 ± 0.44	1.90 ± 0.45	2.02 ± 0.53	2.08 ± 0.60*	2.02 ± 0.34	2.00 ± 0.36	0.790	0.068	0.04

Subgroup A—wearables and personal consultation, Subgroup B—wearables only, Subgroup C—no technical or personal support

*HDL* high density lipoprotein; *BW* body weight

*p < 0.05 visit 2 versus visit 3

^a^p < 0.01 post hoc time × group effect A versus B

Though the work ability (WAI) decreased in subgroup C (no technical or personal support) (Table 3) at visit 3, all subgroups showed a good work ability (37–43 points) after maintenance treatment phase.

**Table 3:** Change in outcomes by questionnaires after 6 months maintenance treatment phase

	A		B		C		Time	Time × group	
	visit 2	visit 3	visit 2	visit 3	visit 2	visit 3	p-value	p-value	η^2^
WAI_total_	38.3 ± 4.8	37.7 ± 6.0	40.2 ± 4.1	40.2 ± 6.2	40.3 ± 5.0	39.1 ± 5.7*	0.115	0.387	0.01
Everyday activity (MET*h/week)	32.0 ± 24.0	25.6 ± 16.8	34.3 ± 23.8	23.7 ± 19.7*	33.5 ± 23.6	25.5 ± 19.3*	< 0.001	0.675	0.01
Exercise activity (MET*h/week)	16.0 ± 16.7	11.5 ± 11.7	18.3 ± 27.0	20.1 ± 36.1	18.0 ± 17.1	11.4 ± 9.8*	0.105	0.178	0.03
Total PA (MET*h/week)	48.0 ± 33.6	37.1 ± 23.0*	52.6 ± 35.7	43.8 ± 40.7*	51.5 ± 29.7	36.9 ± 22.8*	< 0.001	0.685	0.01
Anxiety severity (HADS-D)	3.67 ± 2.40	4.30 ± 3.20	4.31 ± 3.14	4.19 ± 3.64	3.70 ± 3.35	3.88 ± 4.29	0.319	0.419	0.01
Depression severity (HADS-D)	2.77 ± 2.52	3.33 ± 3.08	3.33 ± 3.10	2.98 ± 3.02	2.33 ± 2.51	2.98 ± 3.27*	0.140	0.064	0.04
Physical component score (SF-36)	49.6 ± 7.9	50.0 ± 8.6	50.8 ± 7.6	51.4 ± 9.7	51.7 ± 6.2	51.9 ± 5.6	0.467	0.957	0.00
Mental component score (SF-36)	54.0 ± 5.5	54.0 ± 5.5	52.2 ± 9.1	52.2 ± 9.1	53.9 ± 6.9	53.8 ± 6.9	0.382	0.394	0.01

Subgroup A—wearables and personal consultation, Subgroup B—wearables only, Subgroup C—no technical or personal support

*WAI* work ability index, *PA* physical activity

*p < 0.05 visit 2 vs visit 3

Everyday and total PA (MET*hours/week) showed a decline in all three subgroups with no differences between subgroups (Table 3).

Mental component sum score of health-related QoL (baseline: 49.2 ± 9.5, visit 2: 53.3 ± 7.3, visit 3: 53.2 ± 7.3; p < 0.001, η^2^ = 0.19) increased significantly in the whole intervention across all three time points. Mental component sum score (baseline vs visit 2: p < 0.001, baseline vs visit 3: p < 0.001, visit 2 vs visit 3: p = n.s.) increased between baseline and visit 2 and remained above baseline at visit 3. The physical and mental component sum scores of health-related QoL (SF-36) showed no significant changes over time (Table 3).

Anxiety severity (baseline: 5.37 ± 3.22, visit 2: 3.89 ± 2.98, visit 3: 4.13 ± 3.71; p < 0.001, η^2^ = 0.15) and depression severity (baseline: 4.72 ± 3.32, visit 2: 2.80 ± 2.73, visit 3: 3.09 ± 3.10; p < 0.001, η^2^ = 0.26) decreased significantly for the whole intervention group across all three time points. Anxiety severity (baseline vs visit 2: p < 0.001, baseline vs visit 3: p < 0.001, visit 2 vs visit 3: p = n.s.) and depression severity (baseline vs visit 2: p < 0.001, baseline vs visit 3: p < 0.001, visit 2 vs visit 3; p = 0.471) decreased between baseline and visit 2 and remained above baseline at visit 3. The mean values of both subscales of the HADS-D questionnaire were within the normal range (0–7 points) for all subgroups (Table 3) with no differences between the subgroups.

Participants of subgroup A and B recorded their performed exercise unites with wearable devices, the results are displayed as total exercise units and exercise units per week (Table 4). Both data declined significantly for both subgroups, yet with no difference between the subgroups. The same applies for the daily step count, which was consulted to analyze participants’ everyday activity (Table 4). In average, participants in both subgroups failed to reach the given study task of 150 min of PA per week during the initial intervention phase and the following maintenance treatment phase. However, both subgroups were able to maintain over 80% of their previous performance, comparing maintenance treatment phase with the initial intervention phase (A: 83% ± 45%, B: 82% ± 39%, p = 0.964). There was no significant difference between both subgroups.

**Table 4:** Comparison of exercise parameters of the initial intervention and maintenance treatment phase

	A		B		Time		Time × group	
	Initial intervention	Maintenance treatment	Initial intervention	Maintenance treatment	p-value	η^2^	p-value	η^2^
Total exercise units	73.8 ± 68.3*	60.0 ± 62.0*	84.5 ± 74.5*	64.6 ± 57.9*	< 0.001	0.15	0.499	0.01
Exercise units per week	2.84 ± 2.63*	2.31 ± 2.38*	3.25 ± 2.87*	2.49 ± 2.23*	< 0.001	0.15	0.499	0.01
PA per week (min)	113.3 ± 105.6	115.6 ± 169.5	127.9 ± 101.2*	103.0 ± 88.8*	0.407	0.01	0.320	0.01
Daily step count	8235 ± 3590*	6876 ± 4106*	7429 ± 3091*	5950 ± 3774*	< 0.001	0.21	0.848	< 0.01
Heart rate (bpm)	116.8 ± 12.6	116.1 ± 12.0	114.6 ± 8.6	115.3 ± 10.4	0.977	0.00	0.272	0.02
Adherence goal fulfilled (weeks)	7.05 ± 7.01	6.28 ± 7.29	8.17 ± 7.07*	6.31 ± 6.73*	0.014	0.07	0.303	0.01
Wear time per week (%)	83.5 ± 17.0*	67.1 ± 28.0*	82.9 ± 12.5*	61.9 ± 29.6*	< 0.001	0.31	0.524	0.01

*p < 0.05 initial intervention vs maintenance treatment

There is no data available by wearable devices for subgroup C (no technical or personal support) at visit 3, because participants of this subgroup had to give back their wearable device at visit 2. It is to mention though, that 20 participants of subgroup C (57% out of 35 participants completed V3) have bought an own wearable device during the maintenance treatment phase. After excluding these participants from subgroup C in the final evaluation, the interaction between time and group showed no difference in contrast to the entire subgroup C regarding the analysis of variance with repeated measurements in all observed parameters.

## Discussion

After an initial 6-month telemonitoring-supported exercise intervention in company employees with diagnosed MetS, we divided the participants 1:3 into three subgroups with different support strategies in the maintenance treatment phase. The leading question was to investigate, whether continuous personal support in combination with wearable devices is the superior strategy to help adults maintain their PA. After a maintenance treatment phase of 6 months, we evaluated participant’s compliance to the given task, the anthropometric and cardio-metabolic outcomes as well as work ability, depression severity and health-related QoL by assessments and questionnaires.

It is known that maintaining PA on a high level is a challenging task. Only few interventions report long-term results (≥ 1 year) by showing a positive effect on PA levels, when considering that participation in PA intervention tend to decrease the longer the intervention lasts [22]. Presumed reasons for decreased participation may be the loss of interest, motivation, enjoyment, time and/or perceived benefit [23]. 12 months after the initial intervention has started, our overall dropout rate was low (Fig. 2). This outcome leads to the conclusion that an intervention phase of 6 months with intense personal and technical support is sustainable for at least half a year to keep participant’s motivation on a high level to participate in a program that promotes a healthy lifestyle.

The given task throughout the maintenance treatment phase was to maintain PA on a high level, targeting the recommendation of WHO of 150 min of moderate activity per week. In our investigation we took this task to assess the compliance to the program by taking the amount of recorded PA per week (in minutes) as well as the total amount of weeks, where this task was successfully fulfilled (Table 4) into account. PA per week (in minutes) was found stable in subgroup A (wearables and personal consultation), yet declined in subgroup B (wearables only). Both subgroups maintained over 80% of the minutes of activity they had previously achieved during the initial intervention phase with intense personal and technical support. This finding emphasizes the effectiveness of our maintenance treatment phase and surpasses the findings presented in a recent review by Madigan et al., where 60% to 80% of PA behavior was maintained [15]. However, we did not find a significant difference between subgroup A (wearables and personal consultation) and B (wearables only).

The initial intervention improved participant’s relative exercise capacity (W/kg_BW_) significantly, which is an important component to reduce the relative mortality risk irrespectively of a stated disease [24]. This health-promoting outcome was found stable for subgroup A (wearables and personal consultation) and C (no technical or personal support) after maintenance treatment phase, which suggests that PA interventions do have the ability to promote longer-term health effects.

PA and physical exercise on a regular basis have a positive influence on mental health and QoL both in people who are healthy [25] and people with diseases [26], considering that there is a correlation between MetS and depression [27] as well as MetS and anxiety [28]. In 2016, major depression was the fifth leading cause of years lived with disability [29], which clarifies the importance of addressing mental health in health care programs especially in workplace settings. Our findings at visit 3 for the mental score of QoL (SF-36) as well as depression severity and anxiety severity (HADS-D) remained stable throughout maintenance treatment phase and above baseline values, which allows the conclusion that a telemonitoring-based exercise intervention can significantly improve components of mental health in the long-term.

Previously, we showed that regular PA improves not only physiological and mental parameters but also work ability [16]. To maintain work ability in identified risk groups, WAI is an effective instrument for initiating and controlling occupational prevention measures. Study participants of all subgroups showed good mean work ability by score (WAI) 12 months after the initial intervention has started. A cross-sectional study with teachers confirms our findings by indicating a relationship between work ability and PA in which female teachers with excellent or good WAI had significantly higher levels of total weekly PA [30]. The results at visit 3 suggest (Table 3) that maintaining good work ability is detached from a specific support strategy including further personal consultation after a successfully implemented supervision for 6 months [30].

Our study has strengths and limitations. As mentioned before, some participants of subgroup C (no technical or personal support) have used an own wearable device, for they had to hand in their given study device at visit 2.

A further limitation is that we cannot rule out the possibility of non-tracked activities. This could limit the number of activities recorded and thereby underestimate overall activities for certain individuals. Non-tracked activities could not be included in the evaluation.

## Conclusions

Our data strengthen previous results that a lifestyle intervention supporting PA could be a reasonable resource to reduce MetS related health risk factors, health-care costs, may support productivity related outcomes [7, 31] and help adults to maintain their PA [15]. After analyzing different support strategies in the maintenance treatment phase, our results suggest comparable outcomes for all three subgroups in terms of exercise capacity (W/kg_BW_), components of MetS, as well as health related QoL, anxiety and depression severity and work ability. Most of the health-associated outcomes could be found stable after 6-month maintenance treatment phase, which shows the effectiveness of our intervention. The combination of both personal and technical support during the initial intervention phase and the initial intervention’s duration seem to be the key to long-term success in employees with MetS that lasts for at least another 6 months.

## Data Availability

The datasets used and/or analyzed during the current study are available from the corresponding author on reasonable request.

## Abbreviations

AG: Working group (German „Arbeitsgemeinschaft“)
BW: Body weight
BMI: Body mass index
fig.: Figure
HADS-D: Hospital anxiety and depression scale questionnaire
HDL: High density lipoprotein
IDF: International diabetes federation
MET: Metabolic equivalents of task
MetS: Metabolic syndrome
MetS-z-score: Metabolic syndrome severity score
PA: Physical activity
QoL: Quality of life
SF-36: Short form 36 questionnaire
V2: Visit 2
V3: Visit 3
WAI: Work ability index
WHO: World health organization
